# Impact of Seasonal and Pandemic Influenza on Emergency Department Visits, 2003–2010, Ontario, Canada

**DOI:** 10.1111/acem.12111

**Published:** 2013-04-16

**Authors:** Dena L Schanzer, Brian Schwartz

**Affiliations:** Centre for Communicable Diseases and Infection Control, Infectious Disease Prevention and Control Branch, Public Health Agency of CanadaOttawa, Ontario; Public Health Ontario and the Department of Family and Community Medicine, University of TorontoToronto, Ontario, Canada

## Abstract

**Objectives:**

Weekly influenza-like illness (ILI) consultation rates are an integral part of influenza surveillance. However, in most health care settings, only a small proportion of true influenza cases are clinically diagnosed as influenza or ILI. The primary objective of this study was to estimate the number and rate of visits to the emergency department (ED) that are attributable to seasonal and pandemic influenza and to describe the effect of influenza on the ED by age, diagnostic categories, and visit disposition. A secondary objective was to assess the weekly “real-time” time series of ILI ED visits as an indicator of the full burden due to influenza.

**Methods:**

The authors performed an ecologic analysis of ED records extracted from the National Ambulatory Care Reporting System (NARCS) database for the province of Ontario, Canada, from September 2003 to March 2010 and stratified by diagnostic characteristics (International Classification of Diseases, 10th Revision [ICD-10]), age, and visit disposition. A regression model was used to estimate the seasonal baseline. The weekly number of influenza-attributable ED visits was calculated as the difference between the weekly number of visits predicted by the statistical model and the estimated baseline.

**Results:**

The estimated rate of ED visits attributable to influenza was elevated during the H1N1/2009 pandemic period at 1,000 per 100,000 (95% confidence interval [CI] = 920 to 1,100) population compared to an average annual rate of 500 per 100,000 (95% CI = 450 to 550) for seasonal influenza. ILI or influenza was clinically diagnosed in one of 2.6 (38%) and one of 14 (7%) of these visits, respectively. While the ILI or clinical influenza diagnosis was the diagnosis most specific to influenza, only 87% and 58% of the clinically diagnosed ILI or influenza visits for pandemic and seasonal influenza, respectively, were likely directly due to an influenza infection. Rates for ILI ED visits were highest for younger age groups, while the likelihood of admission to hospital was highest in older persons. During periods of seasonal influenza activity, there was a significant increase in the number of persons who registered with nonrespiratory complaints, but left without being seen. This effect was more pronounced during the 2009 pandemic. The ratio of influenza-attributed respiratory visits to influenza-attributed ILI visits varied from 2.4:1 for the fall H1N1/2009 wave to 9:1 for the 2003/04 influenza A(H3N2) season and 28:1 for the 2007/08 H1N1 season.

**Conclusions:**

Influenza appears to have had a much larger effect on ED visits than was captured by clinical diagnoses of influenza or ILI. Throughout the study period, ILI ED visits were strongly associated with excess respiratory complaints. However, the relationship between ILI ED visits and the estimated effect of influenza on ED visits was not consistent enough from year to year to predict the effect of influenza on the ED or downstream in-hospital resource requirements.

Surveillance of influenza activity in a jurisdiction involves many components. Influenza-like illness (ILI) consultation rates collected through networks of primary care physicians have provided an important indication of influenza activity and are still a major component of influenza surveillance alongside virologic surveillance and other measures.^1^ However, only a small portion of influenza cases are clinically diagnosed as influenza or ILI and ILI only has a modest specificity in predicting an influenza infection.^2^ For this reason, the full burden of influenza on mortality and hospitalization has been estimated statistically for many years^3^–^6^ and thresholds are routinely applied to ILI consultation rates to identify periods of influenza activity.^7^ In general, little is known about the full effect of influenza on the emergency department (ED),^8^ and significant deficits in preparedness for pandemic influenza and other disease outbreaks have been identified for EDs in the United States.^9^ Attempts to assess the relationship between ILI consultation rates and the full burden of influenza are just emerging.^10^–^13^ The recent availability of an administrative database of ED visits^14^ using standardized coding based on the International Classification of Diseases (ICD)^15^,^16^ provides an opportunity to estimate the full effect of influenza on the operations of the ED, as well as to more fully assess ILI consultation rates as an indicator of this burden.

The primary objective of this study was to estimate the number and rate of ED visits attributable to seasonal and pandemic influenza and describe the effect of influenza by diagnostic category groupings, age, and discharge disposition, with the aim of guiding the planning for and management of the ED during periods of high influenza activity. In addition, front-line health care providers use various real-time indicators, such as weekly time series of influenza activity provided by influenza surveillance systems^1^ for resource planning.^17^ As guidance on the interpretation of these indicators for resource planning is currently limited, a secondary objective was to assess the weekly time series of ILI ED visits as an indicator of the real effect of influenza on the number of ED visits.

## Methods

### Study Design

This was an ecological study design based on data from a retrospective record review of fully deidentified data. The earliest statistical estimates of excess mortality associated with influenza were produced by Serfling in 1963.^3^ This method involved estimating a seasonal baseline for weekly mortality (all-cause or pneumonia and influenza) by fitting a sinusoidal function to the mortality data for periods when influenza was not active and then extrapolating to periods of influenza activity and attributing the excess to influenza. The availability of virologic data facilitated the simultaneous estimation of the burden of influenza and other respiratory viruses and the seasonal baseline.^18^ Subsequently the methodology was adapted and validated in many countries,^4^,^19^–^22^ and extended to estimating the hospitalization burden of influenza.^5^,^6^ As these models confirm, the full burden of influenza is significantly underrepresented by the number of cases with either clinical diagnosis or laboratory confirmation of influenza. As a result, the World Health Organization (WHO) has recommended that the full burden of the pandemic be assessed using statistical methods similar to those used to assess the burden of seasonal influenza.^23^

### Study Setting and Population

Records for patient visits to EDs in the province of Ontario, Canada, were extracted from the Canadian Institute of Health Information (CIHI) patient-specific National Ambulatory Care Reporting System (NACRS)^14^ database for the period of September 2003 to March 2010. Population denominators were obtained from Statistics Canada census and intercensus population estimates.^24^ With a population of 13 million in 2009, Ontario is the largest province in Canada and the only province that fully participated in NACRS over the study period.

### Study Protocol

The International Classification of Disease, Tenth Revision, Canadian version (ICD-10-CA),^15^ was used for chart abstraction. ED visits were stratified by age and diagnostic category and aggregated to weekly levels. All 10 diagnostic fields were assessed to identify patient visits with specific clinical diagnoses. Diagnostic code groupings were chosen based on their association with either influenza symptoms or complications of an influenza infection in numbers likely to be large enough to be estimable. As virologic results were not captured in the NACRS database, any ED visit with a clinical diagnosis of influenza or ILI received an ICD-10 code of J11 (virus not specified). A range of other diagnoses is also possible for patients presenting to the ED due to complications resulting from an influenza infection; an unspecified viral respiratory infection (B34.9, B97.8, and J06.9); or any other acute respiratory infection (J00-J22), a category that includes pneumonia (J13-J18). The effect of influenza on otitis media (H65-H67) was also assessed, as this condition has been associated with influenza infections.^25^ In some cases, a diagnosis of an acute respiratory infection may have been missed or omitted, so the effect of influenza on chronic respiratory visits and the specific diagnostic categories of asthma (J45)^26^,^27^ and chronic obstructive pulmonary disease (COPD; J44),^21^,^28^ which are considered risk factors for influenza complications, were also considered. ED visits without respiratory complaints (J00-J99) were analyzed separately to assess any potential effect of increased patient load due to influenza on the operations of the ED.

While viral identification data^1^ have traditionally been used as the proxy variable for influenza activity in this study design,^6^,^18^ increased use of laboratory testing of hospitalized patients with suspected influenza infections, combined with the recent introduction of ICD-10 for chart abstraction, provides an alternative measure of influenza activity. The use of admissions with laboratory confirmed influenza as the proxy variable for the weekly level influenza activity has been shown to improve model fit and provide better face validity especially during the 2009 pandemic period.^29^ Hence, hospital admissions with identified influenza viruses (ICD-10 codes J10 and J09 for the H1N1/2009 pandemic strain) were extracted from CIHI's Discharge Abstract Database^30^ and used as a proxy variable for the level of influenza activity. Hospital admissions coded to J12.1 (viral pneumonia due to respiratory syncytial virus [RSV]) were used as a proxy for RSV activity. Estimates of absenteeism rates due to seasonal and pandemic influenza^31^ were used as the numerator to calculate the rate ratio for influenza-attributed workplace absenteeism to influenza-attributed ED visits.

### Data Analysis

A regression model similar to the one used for other estimates of the influenza burden in Canada^6^,^21^,^31^,^32^ was fit to the stratified weekly ED time series using SAS Enterprise Guide 4.1 (SAS Institute, Cary, NC). PROC GENMOD with a Poisson distribution, linear link function, and dispersion parameter specified by:


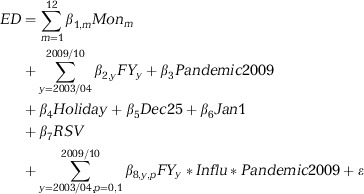


where *ED* represents the weekly number of ED visits for the category of interest (for example, ED visits with ILI diagnoses); the β_1_ parameters account for the baseline seasonality described by monthly indicator variables (*Mon*_*m*_); the β_2_ parameters account for a secular trend with indicator variables for each flu year (*FY*_*y*_), starting in September; β_3_ accounts for any change to baseline ED visits resulting from the declaration of a pandemic; the β_4_, β_5_, and β_6_ parameters account for the effects of holidays, the last week of December, which includes December 25 (Christmas), and the first week of January, respectively; and the β_7_ and β_8_ parameters are multipliers for the proxy variables for RSV (*RSV*) and influenza (*Influ*) respectively. *Pandemic2009* is an indicator variable for the 2009 pandemic period (May 2009–March 2010), *Holiday* is a discrete variable that counts the number of holidays in a week, and *Dec25* and *Jan1* are indicator variables for the last week of December and the first week of January. These variables were included in the model as holiday periods have been shown to be part of the seasonal pattern for hospital admissions.^6^,^29^ Use of the Poisson distribution is recommended when the dependent variable is a count variable corresponding to the number of occurrences or number of items.^33^ The inclusion of a dispersion parameter accounts for excess variation due to events not captured by the choice of explanatory variables.

The number of weekly influenza-attributed ED visits was calculated as the difference between model-predicted visits and the model-predicted visits under the hypothetical absence of influenza (baseline). Confidence intervals (CIs) for estimates of influenza-attributed rates were calculated from the coefficient of variation of the corresponding β_8_ parameter.

The expected background prevalence of symptomatic influenza among persons visiting the ED specifically for nonrespiratory causes was calculated as the average daily ED visit rate for nonrespiratory causes times the duration of clinical illness (2 or 3 days for seasonal and pandemic influenza respectively) times the average annual workplace absenteeism rate as a proxy for the clinical attack rate (11.5% or 13.4%).^31^

## Results

**Figure 1:**
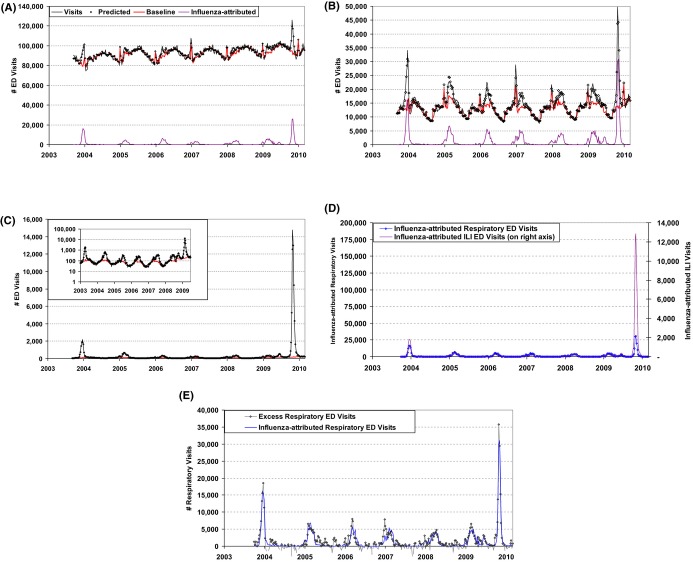
Weekly ED visits, Ontario, Canada, 2004/05–2009/10, NACRS Database, CIHI, showing model fit, estimated baseline, and excess visits attributed to influenza. The estimated baseline curve (*thick red line*) accounts for seasonality and secular trends inherent in (**A**) total ED visits, (**B**) respiratory visits, and (**C**) ILI visits, but in the absence of influenza activity. Model-predicted values (*open diamonds*) correspond closely to the actual number of visits (*thin line*). The excess number of visits attributed to influenza is the difference between model predicted and baseline. As total ED visits are relatively lower during winter months compared to summer months, the excess due to seasonal influenza typically did not correspond to peak visits, unless peak influenza activity aligned with weeks 52 and 1 (Christmas/New Year's period). The 2009 fall pandemic wave was a significant exception, with ED visits increasing to 1.3 times the usual peak levels and influenza accounting for 30% of weekly ED visits at the peak (**A**). H1N1/2009 accounted for only 3% of total ED visits for the pandemic period (May 2009–March 2010). The increase in baseline ILI visits once the pandemic was announced is seen in the log-scale insert (**C**). Seasonal differences between respiratory visits attributed to influenza and influenza-attributed ILI visits are highlighted in (**D**) and the weekly differences between excess respiratory visits (actual – baseline) and respiratory visits attributed to influenza (model predicted – baseline) are shown in (**E**). CIHI = Canadian Institute of Health Information; ILI = influenza-like illness; NACRS = National Ambulatory Care Reporting System.

The estimated rate of ED visits attributable to influenza was elevated during the H1N1/2009 pandemic period at 1,000 per 100,000 (95% CI = 920 to 1,100) population compared to an average annual rate of 500 per 100,000 (95% CI = 450 to 550) population for seasonal influenza. In comparison, rates for ILI ED visits increased from an average annual rate of 55 per 100,000 population to 464 of 100,000 during the H1N1/2009 pandemic. As seen in Figure 1A, total ED visits are generally lower during winter months compared to summer months, and spikes for weeks 52 and 1 are visible. So unless peak influenza activity aligns with weeks 52 and 1 (Christmas/New Year's period in which ED visits normally increase), the excess due to seasonal influenza typically is not associated with a peak in weekly ED visits. The 2009 fall pandemic wave was a significant exception, with ED visits increasing to 1.3 times the usual peak levels, and influenza accounting for an estimated 30% of weekly ED visits at the peak (Figure 1A). Over the entire pandemic period (May 2009 to March 2010), H1N1/2009 accounted for only 3% of total ED visits.

### By Diagnostic Code

Estimates of the rates of ED visits attributed to seasonal and pandemic influenza by ICD-10 diagnostic code grouping are provided in Table 1, which also includes calculations derived from these estimates: the share of all respiratory ED visits attributed to influenza by ICD-10 diagnostic category and the proportion of the annual number of ED visits by category that were attributed to influenza (attributable fraction).

**Table 1:** ED Visits Attributed to Seasonal and Pandemic Influenza, 2003/04–2009/10, Ontario, Canada

	Rate of ED Visits Attributed to Influenza/100,000 Population per Season					Diagnostic Category Proportion of All Influenza-attributed Visits,%§		Attributable Fraction (% Attributed to Influenza)‖	
			
Diagnosis*	Seasonal†	(95% CI)	H1N1/2009†	(95% CI)	Rate Ratio‡	Seasonal	H1N1/2009	Seasonal	H1N1/ 2009
Total ED visits	460	(370 to 550)	880	(670 to 1,100)	1.9	93	86	1	3
Respiratory (J00–J99)¶	500	(450 to 550)	1000	(920 to 1,100)	2.1	100¶	100¶	9	19
Acute respiratory infection (J00–J22)	420	(380 to 460)	970	(870 to 1,100)	2.3	84	94	10	23
Viral respiratory	280	(250 to 300)	790	(730 to 850)	2.9	56	77	17	38
Influenza/ILI (J11)	34	(32 to 36)	390	(380 to 400)	11.5	7	38	58	87
Other viral (B34.9, B97.8, J06.9)	240	(220 to 260)	400	(350 to 450)	1.6	49	38	16	24
Other viral (B34.9, B97.8)	74	(70 to 80)	170	(160 to 180)	2.3	15	17	20	33
Other viral respiratory infection (J06.9)	170	(150 to 190)	220	(190 to 260)	1.3	34	22	14	20
Other acute respiratory infection including pneumonia	120	(100 to 130)	150	(120 to 190)	1.3	23	15	6	9
Otitis media (H65–H67)	27	(20 to 40)	22	(6 to 38)	0.8	6	2	5	5
Chronic respiratory (J23–J99)	78	(70 to 90)	59	(38 to 79)	0.8	16	6	5	5
Asthma (J45)	11	(6 to 15)	8	(1 to 14)	0.7	2	1	2	2
COPD (J44)	15	(13 to 17)	7	(3 to 11)	0.5	3	1	5	3
Other chronic respiratory NOS	53	(50 to 60)	43	(32 to 54)	0.8	11	4	8	7
Nonrespiratory ED visits	–39	(–120 to 40)	–150	(–230 to –68)	3.8	–8	–15	–0.1	–0.9
Expected decline due to community prevalence**	–18		–38						–0.2

* ICD-10 diagnostic category at varying level of detail. Numbers may not add, as they are independent estimates. Up to 10 diagnostic codes are recorded for each visit. Aggregation is indicated by ICD-10 codes. Note that most category groups are mutually exclusive—that is, other viral categories exclude ILI, otitis media excludes any viral respiratory infection and chronic respiratory excludes any acute respiratory diagnosis.

† Figures have been rounded to two significant digits.

‡ Rate ratio of the estimated rate of ED visits attributed to influenza for the 2009 pandemic period to the seasonal average.

§ Calculated as the ratio of the estimated rate of influenza-attributed ED visits for the specific diagnostic category divided by respiratory ED visits attributed to influenza. The respiratory diagnostic category is the reference category for this calculation.

‖ The ratio of the estimated number of ED visits attributed to influenza to the number of ED visits by diagnostic category.

¶ Reference category.

** Calculated based on workplace absenteeism attributed to influenza as a proxy for community prevalence of influenza.^31^ The estimated drop in nonrespiratory ED visits is larger than expected due to community prevalence of influenza alone for the pandemic period.

COPD = chronic obstructive pulmonary disease; ILI = influenza-like illness; NOS = not otherwise specified.

A clinical diagnosis of influenza or ILI was noted in only 7% (1 of 14) and 38% (1 of 2.6) of the estimated excess visits with respiratory complaints that were attributed to seasonal and H1N1/2009 pandemic influenza, respectively (Table 1). An estimated additional 34 and 22% of the influenza-attributed ED visits were coded to J06.9 (unspecified acute viral respiratory infections) for seasonal and pandemic periods, respectively. The acute respiratory category captured an estimated 84% of seasonal and 94% of pandemic influenza visits. Still, an estimated 16% of seasonal and 6% of pandemic influenza-attributed ED visits were for chronic respiratory conditions without any diagnoses for acute respiratory infections recorded.

Visits given the ILI diagnostic code (J11) were more likely than those given other diagnostic codes to be due to influenza. As noted in Table 1, the ILI diagnostic code (J11) had the highest attributable fraction of all diagnostic categories, with an estimated 58% of annual diagnoses attributable to seasonal and 87% attributable to pandemic influenza. ICD-10 codes for (unspecified) viral infections (B34.9, B97.8, and J06.9) had the second highest attributable fractions estimated at 16% for seasonal and 24% or pandemic influenza.

As for the effect on other ED visits, the weekly level of influenza activity was associated with a statistically significant decline in nonrespiratory ED visits of 0.9% (95% CI = −1.4% to –0.4%) during the 2009 pandemic period (Table 1). As an influenza infection is not expected to provide protection against most other emergencies, we estimated that 0.2% of patients who would have visited the ED during the pandemic period with nonrespiratory complaints might have presented to the ED at a time when they also had respiratory symptoms due to influenza infections.

### Age-specific Rates

Estimates of excess ED visits attributed to seasonal and pandemic influenza by age confirm the predominant use of the ED for influenza infections by younger age groups (Table 2A). Relative to seasonal influenza, persons aged 65 years or older made fewer pandemic-related ED visits, while children and young adults made more. Age-specific differences in the proportion of excess ED visits likely due to pandemic influenza for which an influenza/ILI diagnosis was made were not remarkable (Table 2B). For seasonal influenza, it was noted that RSV contributed to baseline ILI diagnoses, particularly for children less than 5 years of age. The estimated rates of excess ED visits for influenza suggests that approximately 3 and 5% of working-aged adults who were sick enough to stay home from work due to influenza infections visited the ED, or equivalently each ED visit attributed to influenza represented on average 40 seasonal or 20 pandemic influenza cases with symptoms severe enough to stay home from work (calculated as the excess ED rates associated with seasonal and H1N1/2009 pandemic influenza of 305 per 100,000 population aged 20 years and 645 per 100,000 population for 64 years, divided by estimated annual absenteeism rates of 11.5% for seasonal and 13.4% for pandemic influenza^31^ and restated as the reciprocal).

**Table 2A:** Age-specific Rates of ED Visits for Influenza, Ontario, 2003/04–2009/10

	ED Visits With a Respiratory Complaint Attributed to Influenza per 100,000 population				
	
Age Group, yr	Seasonal (Annual Average)	(95% CI)	2009 Pandemic	(95% CI)	Rate Ratio
<5	1,300	(1,000–1,600)	2,800	(2,200–3,500)	2.2
5–9	960	(800–1,100)	3,100	(2,800–3,500)	3.2
10–19	560	(490–630)	2,000	(1,800–2,200)	3.6
20–24	430	(380–470)	1,200	(1,100–1,300)	2.8
25–34	370	(340–410)	830	(750–910)	2.2
35–44	280	(250–300)	620	(550–680)	2.2
45–54	250	(230–270)	520	(470–570)	2.1
55–64	250	(230–280)	300	(240–360)	1.2
65+	400	(360–440)	170	(80–260)	0.4
All ages	500	(450–550)	1,000	(900–1,100)	2.0

**Table 2B:** Age-specific Rates of ED Visits for Influenza, Ontario, 2003/04–2009/10

	ILI* Diagnoses in ED per 100,000 population		ILI Visits Attributed to Influenza per 100,000 Population		Estimated Proportion of All Respiratory ED Visits Likely Due to Influenza That Were Clinically Diagnosed as Influenza or ILI	
			
Age Group, yr	Seasonal, Annual Average	2009 Pandemic	Seasonal, Annual Average	2009 Pandemic	Seasonal, Annual Average,%	2009 Pandemic,%
<5	116	1,344	55	1,109	4	40
5–9	62	1,137	30	997	3	32
10–19	58	813	32	700	6	35
20–24	77	657	40	533	9	44
25–34	67	453	34	344	9	41
35–44	48	310	23	227	8	37
45–54	39	243	18	175	7	34
55–64	35	165	16	104	6	35
65+	41	106	20	54	5	32
All ages	55	464	34	389	7	38

ILI = influenza-like illness.

* ICD-10 J11 code includes a clinical diagnosis of influenza or ILI with or without pneumonia. An ICD-10 J11 code indicates that the influenza virus was not identified.

### Admission to Hospital

Overall, the proportion of completed ILI ED visits that resulted in admission to hospital was slightly lower during the pandemic period than for seasonal influenza, declining from 3.8% to 2.8% of ILI ED visits, for an odds ratio (OR) of 0.75 (95% CI = 0.7 to 0.8). However, this decline was due to age-specific differences in the relative disease burden of seasonal and pandemic influenza. The OR was highest for the 45- to 54-year age group at 2.0 (95% CI = 1.6 to 2.5) and was significantly higher for adults aged 25 to 64 years and unchanged for other age groups (Table 3). The proportion of completed influenza-attributed ED visits that resulted in admission to hospital was also lower during the pandemic period than for seasonal influenza, declining from 5.8% to 3.7% of influenza-attributed ED visits, for an OR of 0.62 (95% CI = 0.60 to 0.68). The higher admission rates among influenza-attributed ED visits than for ILI ED visits (5.8% vs. 3.8% for seasonal influenza) implies a lower acuity among ILI ED visits than for all influenza-attributed ED visits.

**Table 3:** Percentage of ED Visits for ILI Resulting in Admission to Hospital, Ontario, 2003/04–2009/10

	% Admitted*				
	
Age Groups, yr	Seasonal	2009 Pandemic	p-value†	OR	(95% CI)
Influenza/ILI diagnosis (J11)					
< 5	3.2	3.5	NS	1.09	(0.91–1.32)
5–9	1.4	1.5	NS	1.08	(0.77–1.54)
10–19	0.9	1.0	NS	1.09	(0.82–1.48)
20–24	1.1	1.0	NS	0.95	(0.65–1.39)
25–34	0.9	1.4	0.007	1.50	(1.12–2.03)
35–44	1.9	2.4	0.045	1.28	(1.01–1.64)
45–54	2.3	4.6	<0.0001	2.01	(1.61–2.53)
55–64	5.8	8.6	<0.0001	1.52	(1.24–1.86)
65+	20.6	20.1	NS	0.97	(0.85–1.11)
All ages (J11 diagnosis only)	3.8	2.8	<0.0001	0.75	(0.70–0.80)
Excess respiratory ED visits					
All ages (influenza attributed)	5.8	3.7	<0.0001	0.62	(0.60–0.64)

NS = not statistically significant.

* Excludes ED discharge status other than admission to hospital or discharge to place of residence; for example, clients who left without being seen by a physician are excluded from the calculation.

† p-values correspond to the null hypothesis of the OR of 1 for each age group.

### Visit Disposition

The effect of influenza activity on the number of ED visits without respiratory complaints by visit disposition is summarized in Table 4. For periods of seasonal influenza activity, an increase in the number of clients who registered but did not complete their visits was noted, along with a similar decline in the number of completed visits. However, during the 2009 pandemic, the decline in completed visits was considerably larger than the increase in the number of registered clients who did not complete their visits. The negative effect of the pandemic on the number of nonrespiratory ED visits was in proportion to the weekly level of influenza activity.

**Table 4:** ED Visits attributed to Seasonal and Pandemic Influenza, 2003/04–2009/10, Ontario, Canada, By Visit Disposition Status

	Seasonal		Pandemic Period (May 2009–March 2010)	
		
Visit Disposition	Total Annual Visits	Impact of Influenza,* *n* (%)	Total Visits	Impact of Influenza,* *n* (%)
Nonrespiratory				
Admitted to hospital	416,900	–600 (–0.1), NS	389,100	–2,000 (–0.5)
Discharged home	3,195,200	–7,400 (–0.2)	3,032,200	–36,500 (–1.2)
Registered but not seen	159,600	7,800 (4.9)	165,900	14,600 (8.8)
Total	3,771,700	–100, NS	3,587,200	–23,800 (–0.7)
% not seen	4.2		4.6	
% admitted	11.1		10.8	
Respiratory				
Admitted to hospital	62,500	3,100 (5)	64,100	4,800 (7)
Discharged home	619,300	51,100 (8)	644,200	127,000 (20)
Registered but not seen	6,600	640 (10)	6,600	1,100 (17)
Total	688,300	54,900 (8)	714,900	133,000 (19)
% not seen	1.0	1.2	0.9	0.9
% admitted	9	6	9.0	3.6

NS = not statistically significant.

* All estimates of the effect of influenza are statistically significant at the 0.01 level, unless flagged (NS). Figures may not add due to rounding.

### ILI as an Indicator of Burden

The difference in the effect of seasonal and pandemic influenza on all respiratory visits compared to ILI visits is illustrated in Figures 1B and 1C. The log scale insert illustrates the seasonality of the ILI baseline and a significant increase in baseline ILI diagnoses throughout the pandemic period. While weekly laboratory confirmed (J10/J09) hospital admissions was a good predictor of excess respiratory and ILI (J11) ED visits (Figures 1B and 1C) based on the specified model, the proportion of excess respiratory visits attributed to influenza that were diagnosed as influenza or ILI was not consistent from year to year (as illustrated in Figure 1D). In fact, the ratio of influenza-attributed respiratory visits to influenza-attributed ILI visits varied from 2.4:1 for the fall H1N1/2009 wave to 9:1 for the 2003/04 influenza A(H3N2) season and 28:1 for the 2007/08 H1N1 season. Excess respiratory visits (actual – baseline) could be monitored as an indicator of the full influenza burden on ED visits, although this time series is sensitive at times to outbreaks due to other respiratory viruses (Figure 1E).

## Discussion

This study confirms a significant effect of both seasonal and pandemic influenza on ED visits. As expected based on results from previous studies of the morbidity and mortality burden of influenza,^6^,^34^ ED visits with clinic diagnoses of influenza or ILI account for only a small portion of the estimated number of influenza-attributed ED visits. In addition, the proportion of excess ED visits attributed to influenza that were clinically diagnosed as influenza or ILI was not consistent from season to season and was considerably higher for the H1N1/2009 pandemic than for seasonal influenza (as illustrated in Figure 1D). The Canadian influenza surveillance system, *FluWatch*^1^ reported unusually high ILI consultation rates (physician visits per 1,000 patient visits) during the pandemic period as well. The higher proportion of ILI diagnoses among ED visits attributed to influenza during the pandemic may be related to a combination of factors: a lower threshold for patients seeking medical care during the pandemic compared to a normal influenza season due to fear of severe illness and increased identification of ILI by physicians in patients with nonspecific illness due to raised index of suspicion.^12^ Excessive use of the ED during the pandemic period was not apparent in this study, although this phenomenon has been identified elsewhere.^35^ Our analysis suggests that the latter, combined with differences in the virulence of the individual strains and the conformity of clinical symptoms to the ILI definition, likely contributed most to variation in the clinical diagnosis of ILI among persons presenting because of influenza infections. Complications often associated with influenza such as otitis media and asthma had low attributable fractions for influenza, and these weekly time series had distinct patterns not captured by the model, which suggests that viruses other than influenza are also responsible for a substantial portion of the morbidity.

While influenza is not typically associated with the peak or surge in total ED visits unless peak influenza activity occurs over the Christmas/New Year's period, an increase in the number of clients with nonrespiratory complaints who registered but did not complete their visits was found to be associated with higher levels of influenza activity in the community. During the pandemic, the effect on visits for nonrespiratory complaints was more pronounced. At peak, ED visits increased to 1.3 times the usual peak levels, and influenza accounted for an estimated 30% of weekly ED visits at peak. The surge during the fall pandemic wave was more concentrated, with 30% of cases occurring during the one peak week versus 15% to 20% for seasonal influenza for seasons in which a single strain circulated.^36^ These findings are not trivial, as “leaving without being seen” is a measure of ED overcrowding, which has been associated with delays to time-dependent therapies and mortality.^37^–^39^

Our estimated rates for ED visits for influenza were similar to those of another study in New York City using similar methods, although we found less variation in rates from season to season.^40^ Aguirre and colleagues^41^ confirm the similarities in presentation between H1N1/2009 and seasonal influenza. Watts and colleagues^7^ report that approximately 41% of all ILI cases from sentinel general practices were confirmed to have influenza infections. In comparison, our estimate was 58% for ED visits with ILI diagnoses. While Metzger and colleagues^10^ estimated that every ED visit for flu-like illness represented approximately 60 illnesses among city residents, our calculations suggest that this relationship is varied. We used estimates of absenteeism attributed to influenza^31^ to calculate that each ED visit attributed to influenza represented on average 40 seasonal or 20 pandemic influenza cases with symptoms severe enough to stay home from work. Noting that the true clinical attack rate of influenza is likely higher than the true absenteeism rate due to influenza, these results are in reasonable agreement. The percentage of ILI visits resulting in admission to hospital was similar for seasonal and H1N1/2009 pandemic influenza for most age groups and slightly elevated for individuals aged 25 to 64 years. However, the percentage of ILI visits that result in admission to hospital is relatively low. Boyle and colleagues^42^ and Sills and colleagues^11^ noted as well that most ED patients with ILI symptoms were discharged home and recommended that special consideration be given to the health service delivery management model for influenza. Keeping in mind that the effect on in-hospital resources of a more virulent pandemic strain is untested, although estimates are available,^43^ and that even in 2009 critical care settings resources were often stretched,^44^ a number of alterative care models have been used during the 2009 pandemic to address this, although further evaluation is still needed. First, during the 2009 pandemic some jurisdictions enhanced primary care services, either within the existing system or by opening targeted influenza assessment, treatment, and referral centers. However, for seasonal influenza, a robust real-time surveillance system is required to detect increases in visits to primary care providers or EDs with enough advance notice to have these special clinics up and running during periods of peak influenza activity. Second, many jurisdictions used telephone consultation programs (with physician- and nurse-staffed help lines) and on-line self-administered screening for ILI. This telephone consultation program resulted in rapid access to antiviral treatment for ambulatory patients, and accurate triage of callers requiring face-to-face assessment, and was well accepted by the population.^45^ A telephone triage tool used during the 2009 pandemic in an obstetrics unit of an urban tertiary care medical center in the United States reduced the volume of in-person encounters and maintained overall good health outcomes.^46^ A similar on-line self-triage tool, which did not include direct access to antivirals, was used in Ontario^47^ and in the United States,^48^ but the effect has not been measured. Finally, pandemic planning exercises have considered innovative models of assessment and prescription of antiviral medication (e.g., nurses, pharmacists, trained providers at telephone triage centers) for severe pandemics or for remote locations when traditional health care provider staffing is limited.

## Limitations

Our approach to estimating the full burden of influenza has provided considerable insight not available using other methods, although this ecologic study design has several limitations. The main uncertainty stems from the use of proxy variables for the level of activity associated with influenza and other respiratory viruses and the ecologic nature of the study design. Earlier studies used weekly virologic data (influenza- or RSV-positive tests or percent positive) as proxies for the weekly level of influenza and RSV activity. With the introduction of ICD-10 coding, we can now replace the proxies based on virologic data with the number of laboratory confirmed influenza or RSV admissions to hospital,^29^ an option that improves the quality of the measures of weekly influenza and RSV activity. CIs were adjusted for the added uncertainty from any factors not captured by the model by including a scale parameter in the regression model.^33^ Although the estimated CIs should be suitable for the study population, generalization to other populations would introduce additional uncertainty. While limitations inherent with an ecologic study design are applicable to this study, multiple studies using this approach for the estimation of the influenza burden suggest that uncertainties are reasonably stated, and the use of statistical models has recently been recommended by the WHO as the preferred option for the estimation of the influenza burden. In most cases, an influenza infection would not protect against other emergencies, and a patient could arrive in the ED with both a nonrespiratory complaint and an unrelated acute influenza infection. While we estimated the background prevalence of influenza in the community to account for these events, the absenteeism rates used in this calculation likely underestimated the true clinical attack rate.

## Conclusions

In this study, influenza had a larger effect on ED visits than was captured by clinical diagnoses of influenza or influenza-like illness, and the effect of these additional ED visits during periods of peak influenza activity was associated with an increase in the proportion of persons who registered but left without being seen. In addition, rates of ED visits attributed to influenza were highest for infants and children, while the proportion admitted to hospital was highest for persons aged 65 years or older. The effect described in this study suggests that there is potential for improvement with alternative health service delivery management models for influenza. Some models, however, would rely on a robust real-time influenza surveillance system that would provide enough advance notice to set up these specialized clinics. Throughout the study period, influenza-like illness ED visits were strongly associated with excess respiratory complaints, and these data may prove to be a useful component of such an alert system. However, the relationship between influenza-like illness ED visits and the estimated effect of influenza on ED visits was not consistent enough from year to year to predict the size of effect of influenza on the ED or downstream in-hospital resource requirements.
